# No Words for Feelings? Not Only for My Own: Diminished Emotional Empathic Ability in Alexithymia

**DOI:** 10.3389/fnbeh.2020.00112

**Published:** 2020-09-11

**Authors:** Elif Alkan Härtwig, Sabine Aust, Hauke R. Heekeren, Isabella Heuser

**Affiliations:** ^1^Department of Education and Psychology, Freie Universität Berlin, Berlin, Germany; ^2^Department of Psychiatry, Charité University Medicine, Campus Benjamin Franklin, Berlin, Germany

**Keywords:** alexithymia, fMRI, cognitive empathy, emotional empathy, subjective arousal

## Abstract

The present study has been designed to disentangle cognitive and emotional dimensions of empathy in a group of mentally healthy and highly alexithymic individuals (ALEX, *n* = 24) and well-matched controls (*n* = 26) through questionnaire Interpersonal Reactivity Index (IRI) and Multifaceted Empathy Task (MET) used during the fMRI and after the fMRI. Simultaneously, Skin Conductance Response (SCR) has been acquired as an implicit measure of emotional reaction. Results show an impaired emotional empathic ability in alexithymic individuals, with lower levels of SCR and higher activation in prefrontal brain regions such as the ventrolateral prefrontal cortex (VLPFC) and inferior frontal gyrus (IFG). Cognitive empathy was not impaired in the alexithymic group and the results were accompanied by a higher activation left IFG. The study leads to the conclusion that alexithymia does not only involve a diminished ability to identify and describe one’s own emotions. Furthermore, it is related to a deeper disability of emotion regulation, which becomes visible through impaired emotional concern for others and higher levels of personal distress.

## Introduction

Understanding each other, cognitively as well as emotionally, is one of the marks of the human. However, humans are differently equipped with the ability to understand others and themselves. Some of these inabilities might stem from a mental disorder, while others can be attributed to differences in personality. Some personality traits lay on the border of personality accentuation and disorder and have yet to be researched. Alexithymia is one such trait.

Alexithymia (Sifneos, 1973) is a personality trait associated with impairments in identifying and describing one’s own emotions, an externally oriented thinking style, and a restricted ability to fantasize. 10% of the general population has been estimated to be highly alexithymic (Franz et al., 2008). Alexithymia has been associated with many psychiatric disorders and is considered a risk factor for mental health (Taylor, 2004; Conrad et al., 2009).

Since the understanding of one’s own emotional states is impaired in alexithymia, there has been growing interest in alexithymia’s implications for social cognition (Grynberg et al., 2010; van der Velde et al., 2013). A core aspect of social cognition, empathy, has been reported to be reduced in alexithymic individuals (Moriguchi et al., 2007; Grynberg et al., 2010). Moriguchi et al. (2006) reported lower levels of emotional concern, the poorer ability of theory of mind (ToM), and higher levels of personal distress in highly alexithymic individuals.

Empathy is an isomorphic affective state arising from the affective state of another individual, in which the observer is aware that the origin of the emotion is within the other (Engen and Singer, 2013). It is a multi-dimensional concept (Davis and Association, 1980; Davis, 1983; Dziobek et al., 2008) with cognitive and emotional dimensions, both separable yet related to each other. According to Dziobek et al. (2008), cognitive empathy (CE) encompasses the capacity to infer affective states of others and to be able to take the perspective of others and, as a result of this process, to be able to name those emotions, a concept similar to Theory of Mind (ToM). Emotional empathy (EE), on the other hand, is one’s emotional response to the emotional states of others, e.g., feeling concern.

Alexithymia is a determining variable of empathic ability in high-functioning autistic individuals (Bird et al., 2010). In a population of autistic spectrum disorders, Dziobek et al. (2008) demonstrated that high-functioning autistic individuals showed lower levels of CE but equal levels of EE compared to people without autism. While it was long assumed that autistic individuals have diminished empathic ability (Gillberg, 1992), research in the last two decades has shown that ToM ability is diminished in high-functioning autistic individuals, while the emotional empathic ability is not (Dziobek et al., 2008). In an earlier fMRI study focusing on interoceptive awareness in individuals with an autism spectrum condition with and without alexithymic symptoms, Silani et al. (2008) suggested that it was the degree of alexithymia, not the autism spectrum condition, that was a stronger predictor of brain activity in the interceptive cortex (i.e., anterior insula) and reduced levels of empathy. Bird et al. (2010) confirmed this when they found increased activation in the left anterior insula while empathizing with the suffering of others. The signal in the left anterior insula was predicted by the level of alexithymia in both the autism spectrum and control groups, hence their suggestion that the level of alexithymia is a predicting factor for the level of empathic ability, not solely autism.

The assumption of the two distinct modes of social cognition also finds support in brain research, with different neural routes for its two components. The Theory of Mind is associated with the medial prefrontal cortex, superior temporal sulcus, and adjacent temporoparietal junction (TPJ; Frith and Frith, 2006; Mitchell, 2008). Emotional empathy is associated with insular, anterior cingulate, and somatosensory cortices (Lane et al., 1998; Bird et al., 2010). Core regions for empathy such as the temporal-parietal junction, temporal pole, orbitofrontal cortex (OFC), and inferior frontal gyrus (IFG) have rarely been reported in studies on alexithymic samples (Kano et al., 2003; Kano and Fukudo, 2013; Moriguchi et al., 2007). As rare evidence, in correlational studies, Moriguchi et al. (2006, 2009) reported impaired IFG and medial prefrontal cortex (MPFC) activity related to alexithymia in a ToM task (Moriguchi et al., 2006).

The current study has been designed to gain a deeper understanding of alexithymia and the cognitive and emotional components of empathy in a mentally healthy sample. As stated above, many studies to date used alexithymia as a correlational variable or used clinical or samples that were not screened. Mental illnesses and their etiological backgrounds are highly complicated. It is a challenge to discern if the ability to identify and describe one’s own emotions is altered due to a previous mental illness or if the alexithymic personality accentuation makes one prone to developing mental disorders. For these reasons, the present study eschews a correlational approach. It is rather based on two extreme groups of high-alexithymic and low-alexithymic individuals, both free of current and past mental illness. This attention to the subjects was not only a methodological issue but is essential to understanding the true relationship between alexithymia and any related human ability, without the confounding effects of mental disorders.

Based on Bird et al. (2010) and Moriguchi et al. (2006), our first hypothesis is that highly alexithymic mentally healthy individuals have an impairment of the emotional dimension of empathy. Guided by the common definition of alexithymia (Sifneos, 1973), alongside an inability of identifying one’s own emotions, our second hypothesis is that subjects with alexithymia suffer from an impairment on the cognitive dimension of empathy.

Research on alexithymia and human brain function has been frustratingly inconsistent. Despite numerous neuroimaging studies on alexithymia (SPECT, fMRI, EEG; PET; see for details: Moriguchi and Komaki, 2013; van der Velde et al., 2013), after more than 20 years of research on brain function in alexithymia, researchers have failed to identify a common pattern of brain function related to altered empathic ability in alexithymia. Thus, our study applies a whole-brain approach without specific ROI-analyses to explore the differences in brain activation patterns in two dimensions of empathy in highly alexithymic individuals and controls. Even though we expect diminished brain activity in core regions of empathy such as the insula, TPJ, prefrontal cortex, IFG and OFC in highly alexithymic individuals (Frith and Frith, 2006; Moriguchi and Komaki, 2013; Decety, 2015).

## Materials and Methods

### Sample

Twenty-four mentally healthy and highly alexithymic individuals (ALEX) from a community based healthy sample and 26 strictly matched control subjects participated in the final sample of the study. All subjects were recruited *via* an announcement on the public transport system in Berlin, Germany. They filled out an online version of the Toronto Alexithymia Scale (TAS-20, Bagby et al., 1994). Those that had a score above 56 and below 40 were invited for further investigation (the cut-offs were decided based on Bagby et al., 1994; Taylor et al., 2003). In the next session, participants filled out a set of questionnaires including the Bermond and Vorst Alexithymia Questionnaire (BVAQ, Vorst and Bermond, 2001). Later on, the participants who were interested in participating and suitable for magnetic resonance imaging were interviewed with a Mini-International Neuropsychiatric Interview (M.I.N.I., Sheehan et al., 1998) clinical interview. All participants with past or current psychiatric and neurological disorders, substance abuse, or severe medical conditions were excluded from the study (see Table 1 for demographics). From the initial of a sample of 60 participants, data of some had to be excluded due to quality reasons of the fMRI-analyses as followed: four controls and three ALEX due to excessive motion, one ALEX because of a lesion, two controls due to their difficulties pressing the buttons and reading the task. Therefore, the final sample is constituted of 24 ALEX and 26 Controls. The local ethics committee of Charité Universitätsmedizin Berlin, Germany approved the study.

**Table 1:** Descriptives and TAS-20, BVAQ and OAS scores of ALEX and Controls.

	ALEX *n* = 24 (11 females)	Controls *n* = 26 (11 females)	*T*-test	
	Mean (SD)	Mean (SD)	*T* (*df*)	*p*
Age	34.96 (10.52)	34.69 (10.05)	0.091 (48)	0.928
Years of education	12.83 (0.63)	12.46 (1.33)	1.271 (48)	0.221
**TAS-20 Total**	64.73 (6.12)	37.54 (4.49)	18.00 (48)	<0.001
DIF	62.20 (10.83)	34.42 (7.55)	10.58 (48)	<0.001
DDF	80.41 (11.02)	39.00 (8.12)	15.20 (48)	<0.001
EOT	57.16 (2.65)	40.12 (7.32)	5.76 (48)	<0.001
**BVAQ Total**	133.20 (14.80)	84.73 (13.38)	12.16 (48)	<0.001
Verbalizing	33.58 (4.35)	15.96 (4.05)	14.82 (48)	<0.001
Fantasizing	22.41 (6.73)	19.19 (6.68)	1.69 (48)	<0.001
Identifying	28.20 (5.16)	13.96 (4.19)	10.74 (48)	<0.001
Emotionalizing	26.08 (3.32)	21.15 (3.36)	5.20 (48)	<0.001
Analyzing	22.91 (7.05)	14.46 (4.34)	5.14 (48)	<0.001
**OAS**	***n* = 21 (8 females)**	***n* = 20 (7 females)**	
OAS Total	1.16 (0.40)	0.77 (0.19)	3.920 (39)	<0.001
Distant	1.58 (0.42)	0.94 (0.40)	4.935 (39)	<0.001
Insightful	0.98 (0.51)	66 (0.32)	2.420 (39)	0.021
Humor	0.95 (0.50)	0.57 (0.47)	2.475 (39)	0.018
Rigid	1.22 (0.70)	0.71 (0.49)	2.479 (39)	0.009
Somatization	0.77 (0.65)	0.89 (0.52)	2.718 (39)	0.529

*Abbreviations: TAS-20, Toronto Alexithymia Scale; DIF, Difficulty Identifying Feelings; DDF, Difficulty Describing Feelings; EOT, Externally Oriented Thinking; BVAQ, Bermond Vorst Alexithymia Questionnaire; OAS, Observer Alexithymia Scale*.

### Procedure

fMRI study was a part of broader investigations on alexithymia, which were conducted in several sessions. Assessment of alexithymia and the psychiatric interview were employed in the preceding sessions. On the day of the experiment, current depressive mood and state and trait anxiety were assessed just before the experiment. Participants were informed thoroughly about the experiment and magnet imaging. A demo version of the experiment (with other stimuli than in the original experiment) was shown on a PC. Participants gave written consent before the experiment and were reimbursed with 20 Euros at the end of the session.

After the acquisition of imaging data, subjects participated in a post-experimental rating on a PC. The post-Experimental rating was identical to the experiment (see “Stimuli and Task” section) but with a Likert scale of 1–9 and there was an additional block for “the experienced clarity of the seen emotion” with the question: “How well does this picture depict the emotion X?”

### Psychometric Assessment and Analyses

The level of alexithymia was measured thoroughly by TAS-20, BVAQ, and Observer Alexithymia Scale (OAS, Haviland et al., 2000). Alexithymia is associated with anxiety (Espina Eizaguirre et al., 2004) and depression, therefore the Spielberger State-Trait-Anxiety-Inventory (STAI, Spielberger et al., 1983; Laux et al., 1991) and Beck Depression Inventory (BDI; Beck et al., 1961) were examined to control for possible confounding variables. Additional to the exclusion of all patients with past and current mental disorders, participants with a BDI score over 18 were excluded from further analysis (Beck et al., 1988).

### Measures

#### TAS-20

Twenty-Item Toronto Alexithymia Scale is a self-report instrument with replicated validity and reliability (Bagby et al., 1994). It gives a total score and scores from three subscales; Difficulty Describing Feelings (DDF), Difficulty Identifying Feelings (DIF), and External Oriented Thinking (EOT); i.e., focusing on external and technical dimension of a theme rather than focusing on feelings or other aspects of inner experience.

#### BVAQ

The Bermond-Vorst-Alexithymia-Scale (Vorst and Bermond, 2001) consists of five scales, each scale comprising eight items. It is one of the measures of alexithymia with high statistical characteristics. It was developed in Dutch and validated in many other languages, including German. The scales of BVAQ are Emotionalizing, Fantasizing, Identifying, Analyzing, and Verbalizing.

#### Observer Alexithymia Scale (OAS)

The Observer Alexithymia Scale (OAS, Haviland et al., 2000) is a third personal rating of alexithymic dimensions by a close friend or relative of the person. OAS was developed based on the Q-Sort technique, adjective descriptions of scientific and clinical experts of alexithymia. It has good internal consistency and high correlations with self-report alexithymia scales (Haviland et al., 2001). It reveals a total score and six subscores.

#### Interpersonal Reactivity Index (IRI)

Empathic ability was assessed using the Interpersonal Reactivity Index (IRI, Davis and Association, 1980; Davis, 1983), which consists of four subscales: Perspective Taking*; i.e., cognitively taking the perspective of the other*, Empathic Concern*; i.e., being emotionally concerned for the other*, Personal Distress; *i.e., experiencing negative feelings in response to other people’s distress* and Fantasy*; i.e., emotional identification with fictional figures*.

#### Statistical Analyses of Questionnaire Measures

Differences between groups on all questionnaire measures and demographical paradigms (except gender) have been analyzed through *T*-test with separate groups analysis or analysis-of-variance (ANOVA) using SPSS-25 (IBM Corp., 2017).

## fMRI Study

### Stimuli and Task

**Figure 1:**
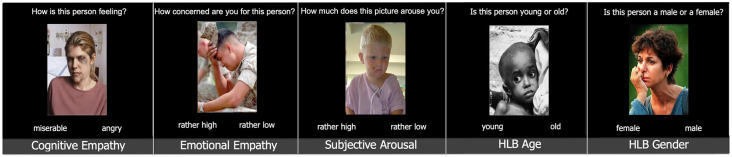
Tasks of the experiment [a modified version of multifaceted empathy task (MET), Dziobek et al., 2008]. Stimuli were presented in blocks of 10. Abbreviations: HLB, high-level-baseline.

A modified fMRI adaptation of the Multifaceted Empathy Task (MET; Dziobek et al., 2008) was used as an experimental task (see Figure 1 for the details). The task included 40 pictures of faces with the emotional expression of negative valence. In all conditions accompanying the emotional picture, a question was presented with a dichotomous answer. Please note that after the fMRI experiment the subjects answered the same questions as in the experimental conditions again with a Likert-Scale of 1–9 during the post-experimental ratings on a PC.

There were three experimental conditions, namely: cognitive empathy, emotional empathy, subjective arousal; and two high-level baseline conditions, namely: high-level baseline gender and high-level baseline age:

Cognitive Empathy (naming the emotion of the other): How is this person feeling? (with two emotion adjectives as answer choices).

Emotional Empathy (emotional concern for the other): How concerned are you for this person? (rather high/rather low).

Subjective Arousal: How much does this picture arouse you? (rather high/rather low).

High-level-baseline gender: Is this person male or female? (female/male).

High-level-baseline age: Is this person young or old? (young/old).

A block of just observing the stimuli without responding and another block of just responding to press button commands have been shown once in each experimental run. These blocks were included in the study as extra control conditions.

An extra block for the experienced level of clarity of the emotional stimuli was included in the post-experimental-rating but was not a part of the fMRI-task: “How well this picture depicts the emotion X?” which was rated on a Likert-Scale of 1–9.

### Experimental Procedure

**Figure 2:**
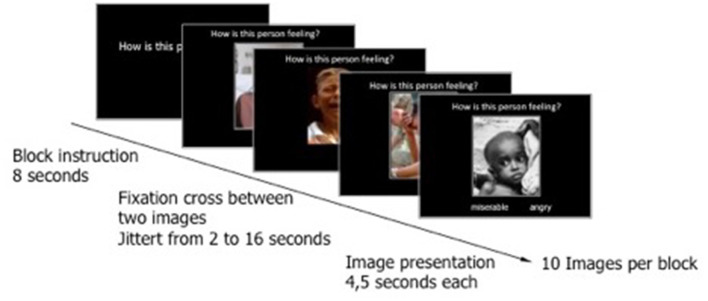
Presentation of an experimental block. Cognitive empathy as an example. Each block has started with a block question and continued with 10 stimuli including a picture depicting a person in a negative emotional state, the typical block question, and the dichotomous answers.

Each block started with the block question (8 s). Each stimulus was presented for 4.5 s. Inter-trial intervals were jittered (minimum 2 s, maximum 16 s, mean 4 s) using OptSeq2 (OptSeq^1^). Stimuli were pseudo-randomized within each block and equally distributed across the blocks (see Figure 2 for the details). The experiment consisted of two runs with 10 blocks in each and the order of the blocks was counterbalanced according to the odd and even ID-Numbers. Each run lasted 13.5 min. There was a short break between the runs. If the participant desired, s/he could wait for several minutes before the second run started. Stimuli were displayed using the experimental control software Presentation (Neurobehavioral Systems Inc., Albany, CA, USA^2^).

The experimental stimuli were presented on the goggles worn by the subjects. The sight was corrected individually for the subjects who needed eyeglasses.

### Data Acquisition

Whole-brain MRI Data was collected on 3 Tesla Siemens Tim Trio (Erlangen, Germany) A scanner with a standard head coil of 32-Channels was used. Head movement was minimized with foam rubber pads. A sagitally oriented T1-weighted structural volume (TE: 2.52 ms; TR: 1,900 ms; flip angle: 9°; FoV: 256; voxel size, 1 × 1 × 1 mm) was acquired for the registration of functional images. Echoplanar data (T2*) was acquired using the standard parameters (TE: 35 ms; TR: 2,000 ms, flip angle: 90°, FOV: 256 mm; matrix: 64 × 64; voxel size, 3 × 3 × 3 mm; 37 slices).

### Data Analysis

The pre-processing of the data was carried out using FEAT from FMRIB’s Software Library (FSL^3^; Smith et al., 2004). Before statistical analysis, the following steps were employed: slice-motion correction using MCFLIRT (Jenkinson et al., 2002), slice-time correction using Fourier-space time-series phase shifting, and non-brain removal using BET (Smith, 2002). The normalized images were smoothed using 8 mm FWHM Gaussian Kernel and were high-pass filtered (sigma = 50.5 s).

FLIRT (Jenkinson and Smith, 2001; Jenkinson et al., 2002) was used for the linear registration of functional images (T2*) to subject’s own high-resolution (T1) and high-resolution image to a standard image implemented by the program (MNI-152). Later on, during the group analysis, these two transformations were combined, which brings the low-resolution functional images (T2*) directly to high-resolution the standard image (MNI-152).

fMRI data were analyzed in a general linear model implemented by FEAT of FSL. Time series were modeled for each individual using event-related regressors for five conditions, instruction, and response (pressing the button) and convolved with the gamma-variate of the hemodynamic response function. The separate baseline conditions for gender and age were aggregated for further analyses and named as HLB (high-level-baseline). Contrast images for Cognitive Empathy (Cognitive Empathy vs. HLB), Subjective Arousal (Subjective Arousal vs. HLB), Emotional Empathy (Emotional Empathy vs. HLB) were computed for each participant. During the group analysis, the functional images of these contrasts were transformed into the standard space (Jenkinson et al., 2002), as explained above. In the higher-level analyses, we reported the activations of cluster corrected (*z* > 2.7, *p* < 0.05) whole-brain data.

### Psychophysiological Data Acquisition and Analyses

Electrodermal activity (EDA) was measured as skin conductance response (SCR) with constant-voltage-technique. We applied electrode paste and placed silver-silver chloride MR capable electrodes (Brain Products Gmbh, Gliching Germany) at the palmar middle phalanges of the index and middle fingers of the left hand. The SCR signal was recorded in DC mode using a bipolar BrainAmp ExG MR amplifier (Brain Products Gmbh, Gliching, Germany).

EDA data was analyzed using BrainVision Analyzer 2 (Brain Products Gmbh, Gliching Germany). Mean amplitude (Max-Min) over all stimuli was used as the main parameter for EDA analyses. First of all, high-pass (5 Hz, 24 dB/oct) and low-pass (0.016 Hz, time constant: 9.947, 24 dB/oct) filters were applied. Then we applied local DC detrend and baseline correction beginning 500 ms before the stimulus presentation. Min and Max markers were put automatically for each stimulus segment and corrected manually by a research assistant, who was blind to the knowledge alexithymia level of the subjects. The absolute difference between the lowest point and highest point of an SCR-curve was transported into SPSS 22 (IBM Corp., 2013) for further analysis. The mean of the amplitude from each stimulus of a block was calculated and used for group comparisons.

## Results

### Descriptive Statistics

There were no differences between the groups concerning age, years of education (see Table 1 for details), and gender (*X*^2_(1)^ = 0.063; *p* = 0.513). The mean age of the 50 participants was 34.8 (SD = 10.17).

The highly alexithymic (ALEX) participants (*n* = 24, 11 females) and the low alexithymic (control) participants (*n* = 26, 11 females) differed significantly on all subscales and total score of BVAQ and TAS-20 (the groups were built according to TAS-20 scores. See “Sample” section and Table 1 for the details).

ALEX had significantly higher scores on OAS-Total and all subscales of OAS but somatization (see Table 1 for the details). OAS was filled out by partners or first-grade relatives of the subjects with subjects’ consent and sent directly to our institutes by the observers. We received the filled-out OAS of 21 ALEX (eight females) and 20 control subjects (seven females).

**Figure 3:**
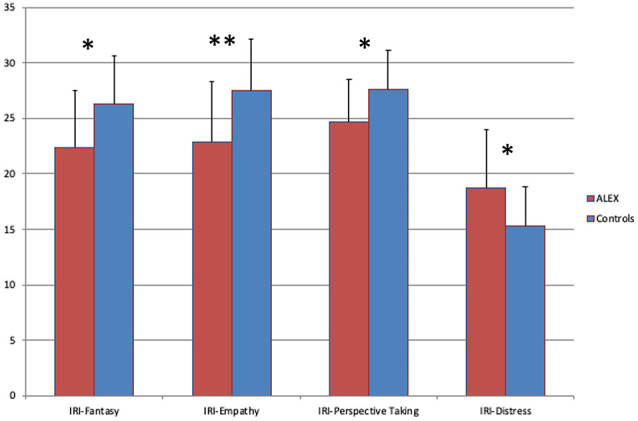
Scores of Interpersonal Reactivity Index (IRI), ALEX had lower scores on Empathy Fantasy and Perspective Taking subscales and higher on Personal Distress subscale. The Error-bars indicate standard deviation (SD). *p < 0.05, **p < 0.001. Abbreviations: ALEX, highly alexithymic individuals.

ALEX had significantly lower scores on all empathy-related subscales of IRI and higher than controls on Personal Distress (see Table 2 and Figure 3 for details).

**Table 2:** IRI, BDI and STAI scores of ALEX and Controls.

	ALEX*n* = 24 Mean (SD)	Controls*n* = 26 Mean (SD)	*T*-test	
			*T* (*df*)	*p*
IRI
Fantasy	22.38 (5.1)	26.31 (4.4)	−2.933 (48)	0.011
Empathy	22.88 (5.5)	27.54 (4.7)	−3.240 (48)	<0.001
Perspective taking	24.71 (3.8)	27.58 (3.7)	−2.688 (48)	0.047
Personal distress	18.71 (5.3)	15.35 (3.6)	2.641 (48)	0.045
Competence	24.25 (2.8)	24.81 (2.4)	−0.751 (48)	0.456
BDI	5.58 (3.10)	2.46 (2.10)	4.12 (48)	<0.000
STAI-State	34.88 (5.76)	31.81 (6.31)	1.78 (48)	0.80
STAI-Trait	37.16 (6.48)	30.26 (4.57)	4.37 (48)	<0.000

*Abbreviations: ALEX, highly alexithymic individuals; SD, standard deviation; IRI, Interpersonal Reactivity Scale; BDI, Beck Depression Inventory; STAI, State-Trait Anxiety Inventory*.

Although clinically insignificantly small, ALEX had higher scores on BDI and STAI-T than controls. Therefore, all further analyses were controlled for depressivity and trait anxiety. The groups did not differ on state anxiety (see Table 2 for details).

### Results From the fMRI Experiment

#### Subjective Ratings of Emotional Experience

**Figure 4:**
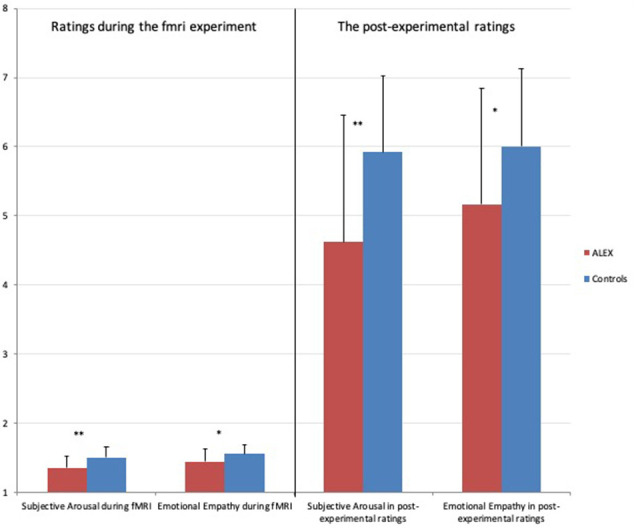
Differences in ratings of subjective arousal and emotional empathy during the fMRI experiment and the post-experiment-ratings. Note: Subjective Arousal during fMRI: ratings of subjective arousal during fMRI experiment range: 1–2, Emotional Empathy during fMRI: ratings of emotional empathy during fMRI experiment range: 1–2, Subjective Arousal in Post-experimental ratings: ratings of subjective arousal in post-experiment range: 1–9, Emotional Empathy in post-experimental ratings: ratings of emotional empathy in post-experiment range: 1–9. The error bars indicate the standard deviation (SD). *p < 0.05, **p < 0.005.

ALEX showed significantly lower emotional empathy and subjective arousal than the controls, which has been seen in the main effect of group in ANOVA (*F*_(1,46)_ = 8.248, *p* = 0.006; controlled for BDI and STAI-T) and in *post hoc* *t*-tests of Empathy-condition (*T*_(48)_ = −2.585; *p* = 0.012) and Arousal-condition (*T*_(48)_ = −3.154; *p* = 0.003; see Figure 4 for details). The main effect of the task and the interaction between group and task were not significant.

When reaction times were taken into consideration, there appeared to be a significant main effect of the task (*F*_(3,138)_ = 7.725; *p* < 0.000), indicating that subjects reacted faster in the HLB-condition than in any other condition (controlled for BDI and STAI-T). The main effect of group and group task interaction was not significant, showing that ALEX was not slower or faster than controls.

### Post-experimental Ratings

There was a significant main effect of task (*F*_(3,135)_ = 5.155; *p* = 0.002). The group task interaction was significant (*F*_(3,135)_ = 6.470; *p* = 0.004). *Post hoc* *t*-tests revealed lower arousal (*T*_(47)_ = −3.000; *p* = 0.004) and emotional empathy ratings (*T*_(47)_ = −2.165; *p* = 0.035) in ALEX.

The main effect of group was not significant. In reaction times, there was a significant main effect of the task (*F*_(3,135)_ = 2.907; *p* = 0.049) indicating slowest responses in the condition of “experienced clarity of the seen emotional expression.” The main effect of group and task group interaction was not significant (all results have been controlled for depressivity and trait anxiety).

#### Electrodermal Activity

EDA data from only nine ALEX and 13 controls are reported. Due to a technical problem (mistaken usage of false electrodes during half of the sample data collection) the data of 28 subjects had to be eliminated from further analysis. The remaining individuals were representatives of the original ALEX and control samples. The remaining samples of ALEX and controls did not differ on depressivity (*p* < 0.103) but trait-anxiety (*p* < 0.000). For this reason, further analyses on EDA have been controlled only for trait-anxiety.

In arousal condition there was a group difference *p* = 0.014 (Mann–Whitney-*U* = 22.000; *Z* = −2,437), indicating lower SCR in ALEX.

#### fMRI Results

##### Main Effects

*Cognitive Empathy (vs. HLB)*. Separate mixed-effects analysis for groups of ALEX and controls for the cognitive empathy condition compared to HLB revealed similar activation in both groups in areas related to social cognition such as left superior temporal sulcus, left TPJ, left IFG, left OFC and left temporal pole (see Table 3 for cluster sizes and coordinates).

**Table 3:** Main effect of cognitive empathy in separate groups.

Main effect of cognitive empathy in ALEX						
		MNI coordinates				
Brain region	H	*x*	*y*	*z*	*z*-score	Volume, mm^3^
Posterior STS/posterior middle temporal gyrus (extending to temperoparietal junction, orbitofrontal cortex and inferior frontal gyrus)	L	−56	−50	4	7.59	602,532
Temporal pole (extending to orbitofrontal cortex)	R	50	−26	−8	5.81	117,072
Superior frontal gyrus	L	2	10	60	5.1	59,940
Precentral gyrus	R	46	2	50	4.23	12,987
**Main effect of cognitive empathy in controls**						
Inferior frontal gyrus (extending to orbitofrontal cortex and STS)	L	−46	18	26	5.53	126,765

*Abbreviations: ALEX: highly alexithymic individuals, MNI: Montreal Neurological Institute, H: Hemisphere, L: Left, R: Right, STS: superior temporal sulcus*.

*Emotional Empathy (vs. HLB)*. Separate mixed-effects analysis for groups of ALEX and controls revealed different patterns of activation in this contrast. The signal change was observed in controls only in the left OFC and IFG. ALEX in addition to those areas above had activation in left orbitofrontal gyrus (OFC), extending to ventrolateral prefrontal cortex (VLPFC), right OFC, right IFG, left TPJ (see Table 4 for cluster sizes and coordinates).

**Table 4:** Main effect of emotional empathy in separate groups.

Main effect of emotional empathy in ALEX						
		MNI coordinates				
Brain region	H	*x*	*y*	*z*	*z*-score	Volume, mm^3^
Orbitofrontal cortex (extending to VLPFC)	L	−48	28	−8	6.02	160,623
Superior frontal gyrus	L/R	−10	28	56	5.25	141,264
Orbitofrontal cortex	R	34	22	−18	4.75	54,621
Angular gyrus (extending to temperoparietal Junction)	L	−42	−60	18	4.57	53,325
Posterior STS	R	52	−28	−10	4.62	26,946
Inferior frontal gyrus pars triangularis	R	48	22	18	4.64	13,446
Precentral gyrus	R	44	4	42	4.2	11,070
**Main effect of emotional empathy in Controls**						
Orbitofrontal cortex (extending to inferior frontal gyrus)	L	−48	18	8	3.77	14,688

*Abbreviations: ALEX, highly alexithymic individuals; MNI, Montreal Neurological Institute; H, Hemisphere; L, Left; R, Right; VLPFC, ventrolateral prefrontal cortex; STS, superior temporal sulcus*.

*Subjective Arousal (vs. HLB)*. The contrast subjective arousal compared to HLB revealed similar brain areas in each group: left TPJ, left IFG, left OFC. Additionally, ALEX had a higher signal change in right IFG, right OFC in ALEX only. Bilateral PCC had higher signal change only in controls in this contrast (see Table 5 for cluster sizes and coordinates).

**Table 5:** Main effect of subjective arousal in separate groups.

Main effect of subjective arousal in ALEX						
		MNI coordinates				
Brain region	H	*x*	*y*	*z*	*z*-score	Volume, mm^3^
Superior frontal gyrus (extending to orbitofrontal cortex and inferior frontal gyrus)	L/R	2	10	62	4.92	174,555
Posterior cingulate cortex, precuneus	L/R	10	−72	8	3.96	54,189
TPJ	L	−48	−60	20	4.13	20,088
IFG pars opercularis (extending to insula)	R	52	22	−4	3.9	17,955
Middle frontal gyrus	L	−44	8	40	3.96	9261
**Main effect of subjective arousal in controls**						
Middle frontal gyrus (exteniding to IFG, orbitofronal gyrus and temporal pole	L	−46	6	48	5.68	96,093
STS extending to TPJ	L	−54	−46	4	4.86	37,395
Superior frontal gyrus extending to anterior cingulate cortex	L/R	0	16	54	4.38	30,105
Posterior cingulate cortex	L/R	−2	−16	32	3.72	11,178
Middle temporal gyrus (extending to TP)	R	52	−10	−16	3.47	10,692
Superior frontal gyrus	L	−2	54	28	4.06	8,856

*Abbreviations: ALEX, highly alexithymic individuals; MNI, Montreal Neurological Institute; H, Hemisphere; L, Left; R, Right; IFG, inferior frontal gyrus; TPJ, Temperoparietal junction; STS, superior temporal sulcus*.

##### Group Effects

**Figure 5:**
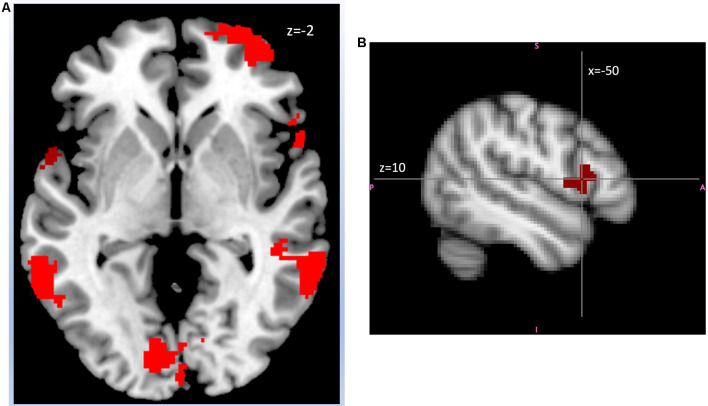
**(A)** ALEX (*n* = 24) showed in right ventrolateral prefrontal cortex (VLPFC), left inferior frontal gyrus (IFG), bilateral middle temporal gyrus, and left precuneus cortex (Montreal-Neurological-Institute Coordinates *z* = −2, Axial image) than controls (*n* = 26) in the contrast cognitive empathy vs. high-level baseline. Highlighted areas indicate a significant difference between groups in blood oxygen level difference (BOLD) signal (cluster corrected *z* > 2.7, *p* < 0.05). Abbreviations: ALEX, highly alexithymic individuals. **(B)** ALEX (*n* = 24) showed higher activity in left opercular-IFG (Montreal-Neurological-Institute Coordinates *x* = −50, *y* = 14, *z* = 10) than controls (*n* = 26) in the contrast cognitive empathy vs. high-level baseline. Highlighted areas indicate a significant difference between groups in BOLD signal (cluster corrected *z* > 2.7, *p* < 0.05). Abbreviations: ALEX, highly alexithymic individuals.

*Cognitive Empathy (vs. HLB)*. In cognitive empathy condition (contrasted to HLB) ALEX had higher activation than controls in right VLPFC, right TP, right OFC, right MFG and left opercular-IFG (see Table 6 for cluster sizes and coordinates and Figure 5 for brain activation).

**Table 6:** Group effects ALEX > Controls.

Cognitive empathy (vs. HLB)						
		MNI coordinates				
Brain region	H	*x*	*y*	*z*	*z*-score	Volume, mm^3^
VLPFC (extending to temporal pole and orbitofrontal cortex)	R	36	54	−2	4.45	51,651
Middle temporal gyrus, temporooccipital part	R	56	−30	−8	4.27	17,604
Middle temporal gyrus, temporooccipital part	L	−64	−52	−2	3.57	12,258
Opercular inferior frontal gyrus	L	−50	16	14	3.81	10,881
Precuneus cortex	L	−2	−74	28	3.27	8,397
**Emotional empathy (vs. HLB)**						
VLPFC	R	34	60	−4	3.89	12,717
Orbitofrontal cortex	L	−40	22	−16	3.68	9,720

*Abbreviations: ALEX, highly alexithymic individuals; MNI, Montreal Neurological Institute; H, Hemisphere; L, Left; R, Right; IFG, inferior frontal gyrus; VLPFC, ventrolateral prefrontal cortex; TPJ, Temperoparietal junction; STS, superior temporal sulcus*.

**Figure 6:**
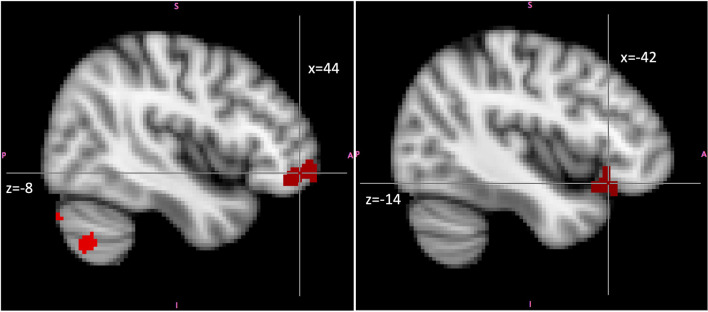
Higher activity in ALEX (*n* = 24) in the contrast emotional empathy vs. high-level baseline in right VLPFC (image on the left; Montreal-Neurological-Institute Coordinates *x* = 44, *y* = 48, *z* = −8) and left OFC (image on the right; Montreal-Neurological-Institute Coordinates *x* = −42, *y* = 24, *z* = −14) than controls (*n* = 26). Highlighted areas indicate a significant difference between groups in BOLD signal (cluster corrected *z* > 2.7, *p* < 0.05). Abbreviations: ALEX, highly alexithymic individuals; VLPF, the ventrolateral prefrontal cortex; OFC, orbitofrontal cortex.

*Emotional Empathy (vs. HLB)*. In emotional empathy condition (contrasted to HLB) ALEX had higher activation in the right VLPFC and left OFC (see Table 5 for cluster sizes and coordinates and Figure 6 for brain activation).

*Subjective Arousal (vs. HLB)*. There were no significant group differences in cerebral brain activation in subjective arousal conditions compared to HLB. The only significant cluster was in the right cerebellum.

##### Task × Group Interactions

There was no statistically significant activation in all Task × Group Interactions.

##### Contrast Cognitive Empathy vs. Subjective Arousal

In contrast, emotion recognition vs. subjective arousal, ALEX showed higher activation in superior temporal gyrus, r-triangular IFG, l-opercular IFG, and bilateral thalamus. In the control group, there was no significant cluster (see Table 7 for cluster sizes and coordinates).

**Table 7:** Contrast cognitive empathy vs. subjective arousal in separate groups.

Main effect in ALEX						
		MNI coordinates				
Brain region	H	*x*	*y*	*z*	*z*-score	Volume, mm^3^
STS	R	−58	−56	4	5.89	186,921
STS	R	60	−52	4	4.82	49,005
IFG pars triangularis (extending to opercular IFG)	R	52	36	8	4.79	12,852
Thalamus	R	2	−10	6	4.09	9,855
Inferior occipital gyrus	R	36	−84	−18	4.3	8,748

*Abbreviations: ALEX, highly alexithymic individuals; MNI, Montreal Neurological Institute; H, Hemisphere; L, Left; R, Right; STS, superior temporal sulcus; IFG, inferior frontal gyrus*.

## Discussion and Conclusion

In this study, we aimed to achieve a deeper understanding of empathic ability in a highly alexithymic sample. For this purpose, we have investigated emotional and cognitive empathy in extreme groups of highly alexithymic (ALEX) and very low alexithymic individuals *via* a questionnaire measure (IRI; Davis and Association, 1980; Davis, 1983) as well as an adapted version of and MET in fMRI, accompanied by measurement of EDA.

Our first hypothesis, that ALEX has an impaired ability of emotional empathy, has been supported by the results of the current study. In the behavioral results of MET, highly alexithymic individuals showed impairment in emotional empathy, both in the explicit measure of emotional concern for others and in the implicit measure of being aroused by the emotional states of others.

The results did not support our second hypothesis that ALEX has an impaired ability of cognitive empathy. Cognitive empathy has been measured by MET, which implemented cognitive empathy as an ability to name the emotional states of others. The present task of cognitive empathy was based on affect labeling, which is crucial for interpersonal communication though is only one aspect of it. ToM, which was not measured in the current study, is another crucial aspect of cognitive empathy and Moriguchi et al. (2006) reported lower ToM in ALEX accompanied by lower activation in MPFC. Hence, our study, by showing an insignificant difference in cognitive empathy, highlights the importance of possible other aspects of interpersonal communication such as ToM. It is important to note that the equal levels of cognitive empathy in the current samples of ALEX and the control group was accompanied by higher activation in several prefrontal brain structures in ALEX, which will be discussed later in this paper.

Following our first and second hypotheses, we expected diminished brain activity in core regions of empathy such as IFG, Insula, MPFC, and OFC. It is important to note that in our study the controls surprisingly never showed a significantly higher activation than ALEX in any region of the brain throughout the tasks, although alexithymia has been associated with diminished brain activity (Taylor and Bagby, 2004; van der Velde et al., 2013; Wingbermühle et al., 2012). On the contrary, both in cognitive and emotional empathy conditions, there has been higher right VLPFC and OFC activation in ALEX. Further, there is evidence linking VLPFC to social cognition (Pinkham et al., 2008) and emotion regulation (Lieberman et al., 2007; Townsend et al., 2012). Activation of VLPFC and simultaneous downregulation of the amygdala has been reported several times (Diekhof et al., 2011; Buhle et al., 2013; Silvers et al., 2016). In a task very similar to our cognitive empathy paradigm, Lieberman et al. (2007) found that right VLPFC specifically down-regulated amygdala activity and reported that enhanced activity in VLPFC might lead to diminished activity of the amygdala, which in turn might be related to declined emotional experience. In a real-time fMRI neurofeedback study Paret et al. (2016) have shown that voluntary down-regulation of the amygdala increased the connectivity between the amygdala and ventromedial PFC, which brings out strong evidence from the first real-time neurofeedback study about the effects of down-regulation between PFC and amygdala. Since we have not employed connectivity analyses we cannot confidently assume that down-regulation of limbic structures *via* higher activation of VLPFC, but it is still a possibility for explaining lower emotional experience in alexithymia, which should be subjected to further research.

In our study, ALEX showed enhanced OFC activation during both empathy tasks. Besides being related to several other cognitive functions, OFC is an important brain structure related to recognizing the significance of emotional stimuli (Levens and Phelps, 2010; Golkar et al., 2012), to emotion regulation (Decety, 2011) and emotional empathy (Cox et al., 2012). Rare studies are reporting altered OFC structure or function in alexithymia. Kano et al. (2003) showed in a PET study decreased regional cerebral blood flow in the right OFC in reaction to negative emotional stimuli in highly alexithymic individuals. van der Velde et al. (2014) report an association between affective alexithymia and lower gray matter volumes in OFC. In a meta-analysis, Xu et al. (2018) report consistently lower gray matter volumes in OFC concerning alexithymia.

According to our findings, in ALEX there has been higher temporal pole activation in both cognitive and emotional empathy tasks. The temporal pole is known to be a region involved in social cognition (Olson et al., 2007) and specifically in the empathy network (Singer, 2006; Frith and Frith, 2011; Shamay-Tsoory, 2011).

ALEX had higher activity in left IFG only in cognitive empathy but not in emotional empathy tasks. Also, in the contrast of cognitive empathy vs. subjective arousal as an implicit measure of emotional empathy, there has been a higher activation in left IFG in ALEX. There is strong evidence showing opercular IFG as a core structure for empathic ability (Moriguchi et al., 2006; Shamay-Tsoory et al., 2009; Fallon et al., 2018), and also for language production (Liakakis et al., 2011; Hobson et al., 2018). IFG’s coexisting contribution to language and emotional processes is coherent with left IFG’s specific involvement in this experiment in a task with more language-related components than subjective arousal tasks, which require the internal representation of one’s own emotional state. Brain areas involved in cognitive and emotional empathy tasks in ALEX were almost the same prefrontal areas, except IFG, which we observed only for cognitive empathy. Prefrontal areas are crucial for the cognitive control of emotion processing (Ochsner and Gross, 2005) and emotion regulation (Lieberman et al., 2007). We assume that the extensive involvement of prefrontal areas may have allowed ALEX compensate their deficits in emotion processing with cognitive empathy (in our experiment conspired as naming emotions/affect labeling) but insufficiently for a positive functioning in emotional empathy, which prerequisites the ability to feel for the other and to be able to recognize congruent feelings in one’s own self.

ALEX reported lower levels of subjective arousal during the fMRI task which was also supported by the implicit physiological measure of lower SCR. At the mean time on the questionnaire measure of IRI (Davis and Association, 1980; Davis, 1983), ALEX reported higher personal distress when they were confronted with others’ discomfort, accompanied by lower levels of empathic concern and cognitive empathy (perspective taking). This result is following the findings of Moriguchi et al. (2006). According to Decety (2011), Eisenberg and Eggum (2009) and Lamm and Tomova (2018), observing another distressed person can result in several different reactions, such as sympathy (also called empathic concern, “feeling for”), emotional contagion “feeling as,” or fear, avoidance, and personal distress. Personal distress is self-directed and aversive and is not aimed at to relieve the other person from uncomfortable feelings but merely one’s own. In contrast, sympathy/empathic concern is an other-directed prosocial feeling. Decety and Lamm (2009) argue that if not regulated, this distress might intervene with an individual’s ability to react and resolve the stressful situation. At this point, it is meaningful to look at stress-related regulation mechanisms. Besides the sympathoadrenomedullary (SAM) system, the hypothalamic-pituitary- adrenocortical-system is the main system of primates responsible for immediate coping with stress through the regulation of cortisol secretion (White and Buchanan, 2016). HPA-system does not react with higher levels of cortisol not only in situations when the individual experience stress herself but also in observing others in distress (Buchanan et al., 2012; Engert et al., 2017). Buchanan et al. (2012) have also reported higher cortisol production by the observants with higher levels of empathic concern and perspective-taking on IRI. These results have also been supported by a recent meta-analysis by Engert et al. (2019). In a previous study of ours (Alkan Härtwig et al., 2013), ALEX have shown lower (HPA) system function measured through cortisol-awakening response (CAR), which indicates an alteration in the basic function of HPA-System in healthy alexithymic individuals even without direct exposure to stress. All these considered, these results point to the possibility of an underlying HPA-system-disfunction in understanding why ALEX show less emotional empathy when confronted with others’ distress.

To our knowledge, this has been the first neuroimaging study that has examined cognitive and emotional empathy simultaneously in alexithymia. The large sample of physically and mentally healthy highly alexithymic participants (ALEX), who were closely matched demographically to their controls, contributed to the strength of the study. Also, the assessment of alexithymia did not only depend on self-report measures but was supported by an observer measurement. Finally, to eliminate all possible interactions with depressivity and anxiousness, all results have been controlled for these two dimensions. Therefore, we are confident that the results of this study reflect alexithymia in and of itself and are not confounded with other psychological problems and disorders, as in many studies using clinical samples.

The current results indicate a specific impairment of alexithymic individuals in experiencing emotions but not in naming them since the ALEX sample showed a similar level of competence in naming emotions as the control group. Experiencing the emotional states is commonly thought to be a precursory of naming the emotions. Still, the current results indicate an impairment in the basic emotional experience which does not directly lead to an impairment in the naming of emotions to the same extent. We propose that higher prefrontal activation overcomes the impairments in emotional functioning in ALEX in basic tasks such as affect labeling. Thus, ALEX participants manage to score similar to controls in cognitive empathy tasks. But this prefrontal activation is insufficient when the tasks involve more complicated functions such as emotional empathy. We also empathize that altered HPA-system function might be also related to lower levels of emotional empathy in ALEX.

How this extensive cognitive effort accompanied by the higher levels of personal distress is related to a dysfunction in the feeling of emotions and if it is due to a possible down-regulation of limbic structures by prefrontal activity (Lieberman et al., 2007; Silvers et al., 2016) should be examined in future studies *via* functional and structural connectivity.

## Data Availability Statement

The datasets generated for this study are available on request to the corresponding author.

## Ethics Statement

The studies involving human participants were reviewed and approved by Ethics Committee of Charitée—Universitätsmedizin, Berlin. The patients/participants provided their written informed consent to participate in this study.

## Author Contributions

EA and HH designed the research. EA and SA collected data. EA analyzed data under supervision of HH. EA, HH, SA and IH wrote the article. All authors read the last version of this manuscript and gave their consent for publication.

## Conflict of Interest

The authors declare that the research was conducted in the absence of any commercial or financial relationships that could be construed as a potential conflict of interest.

## Footnotes

^1^https://surfer.nmr.mgh.harvard.edu/optseq/

^2^www.neurobs.com

^3^www.fmrib.ox.ac.uk/fsl
